# Enhanced Suppression of Immune Cells In Vitro by MSC Overexpressing FasL

**DOI:** 10.3390/ijms22010348

**Published:** 2020-12-31

**Authors:** Ana-Maria Vacaru, Madalina Dumitrescu, Andrei Mircea Vacaru, Ioana Madalina Fenyo, Radu Ionita, Anca Violeta Gafencu, Maya Simionescu

**Affiliations:** Institute of Cellular Biology and Pathology “Nicolae Simionescu”, 050568 Bucharest, Romania; madalina.dumitrescu@icbp.ro (M.D.); andrei.vacaru@icbp.ro (A.M.V.); madalina.fenyo@icbp.ro (I.M.F.); radu.ionita@icbp.ro (R.I.); anca.gafencu@icbp.ro (A.V.G.); maya.simionescu@icbp.ro (M.S.)

**Keywords:** FasL, mesenchymal stromal cells, autoimmunity, immunosuppression, immunomodulation

## Abstract

Mesenchymal stromal cells (MSC) display several mechanisms of action that may be harnessed for therapeutic purposes. One of their most attractive features is their immunomodulatory activity that has been extensively characterized both in vitro and in vivo. While this activity has proven to be very efficient, it is transient. We aimed to enhance it by transforming MSC to overexpress a first apoptosis signal (Fas) ligand (FasL). In this study, our goal was to induce FasL overexpression through adenoviral transduction in MSC to improve their immunomodulatory activity. We characterized the impact of FasL overexpression on the morphology, proliferation, viability, phenotype, multilineage differentiation potential and immunomodulation of MSC. Moreover, we determined their suppressive properties in mixed reactions with A20 cells, as well as with stimulated splenocytes. Our findings demonstrate that FasL-overexpressing MSC exhibit improved immunosuppressive properties, while maintaining their MSC-characteristic features. In conclusion, we establish, in a proof-of-concept set-up, that FasL-overexpressing MSC represent good candidates for therapeutic intervention targeted at autoimmune disorders.

## 1. Introduction

Mesenchymal stromal cells (MSC) display several mechanisms of action that may be exploited for therapeutic purposes [1]. One of their most attractive features for applications targeted at autoimmune disease is their immunomodulatory activity. They hamper the proliferation and function of several major immune cells, such as dendritic cells, T and B lymphocytes and natural killer (NK) cells. This has been characterized by studies, both in vitro and in vivo, where MSC were shown to ameliorate a variety of immune disorders [2,3,4,5,6,7]. Moreover, they induce immune tolerance through upregulation of the CD4+CD25+Foxp3+ population of regulatory T cells (Treg) and suppression of B and T cells [3,8,9].

MSC can be sourced from multiple tissues and organs, such as bone marrow (BM), placenta, adipose tissue, umbilical cord blood and many others. MSC have been defined as fibroblast-like nonhematopoietic progenitor cells with the capacity to differentiate in adipogenic, osteogenic and chondrogenic lineages [10,11,12,13,14,15]. Phenotypically, mouse MSC express CD29, CD51, Sca1, CD105 and CD90 and present the negative expression of hematopoietic CD117 (c-kit), CD45 and CD31 [4,14,15,16,17,18].

The first apoptosis signal ligand (FasL)/Fas interaction embodies the typical caspases cascade signaling pathway involved in the apoptosis of many cell types [19,20,21]. To avoid undesirable cell death, FasL synthesis and exocytosis are tightly regulated at both the transcriptional and post-transcriptional levels [22]. FasL is exposed on the cell membrane for a few minutes from secretion until shedding [23]. The membrane-bound form of FasL and soluble FasL have different actions when bound to the receptor. 

BM-MSC (from here on referred as MSC) express low levels of FasL and induce cell apoptosis [4,9,24]. Moreover, Akiyama and collaborators demonstrated that the endogenous FasL expressed by MSC was able to temporarily increase the apoptosis of T cells via the FasL/Fas pathway, leading to immune tolerance [3]. However, this effect was transient, and for an efficient cure of the disease, the treatment may require repeated infusions of MSC.

To overcome this inconvenience, we proposed to increase the efficacy of MSC by overexpressing the FasL following adenovirus-mediated expression, thus obtaining “killer” MSC. We chose the adenovirus-mediated transduction of the MSC as it is more suitable to infect slow-cycling cells, like MSC [25]. Moreover, it yields a transient expression of the exogenous FasL, as a lentiviral-mediated transduction might have deleterious effects. Therefore, we exploited a naturally occurring process, the suppression of T cells determined by FasL-induced apoptosis, to enhance the immunomodulatory properties of MSC. The impact of FasL overexpression on MSC characteristics (morphology, viability, proliferation, differentiation and phenotype) and immunomodulation potential was tested. In addition, the potential of these modified cells to suppress the immune reaction in vitro in a disease-relevant set-up was determined. We used, in this study, a model for type 1 diabetes (T1D) as a proof-of-concept to demonstrate the functionality of “killer” MSC. We focused our study on T cells, because, apparently, autoreactive T cells are considered the main effectors of β-cell destruction in T1D [26,27,28]. Activated T cells also drive the inflammatory reaction in other autoimmune diseases; thus, the therapeutic approach that we propose here may prove beneficial for various autoimmune diseases as well.

We report here that the characteristic features of MSC were not affected by FasL overexpression, and their immunosuppressive properties were enhanced, becoming more efficient in inducing the death of both A20 cells and stimulated splenocytes.

## 2. Results

### 2.1. Assessment of the Adenovirus-Mediated Overexpression of FasL in MSC

**Figure 1:**
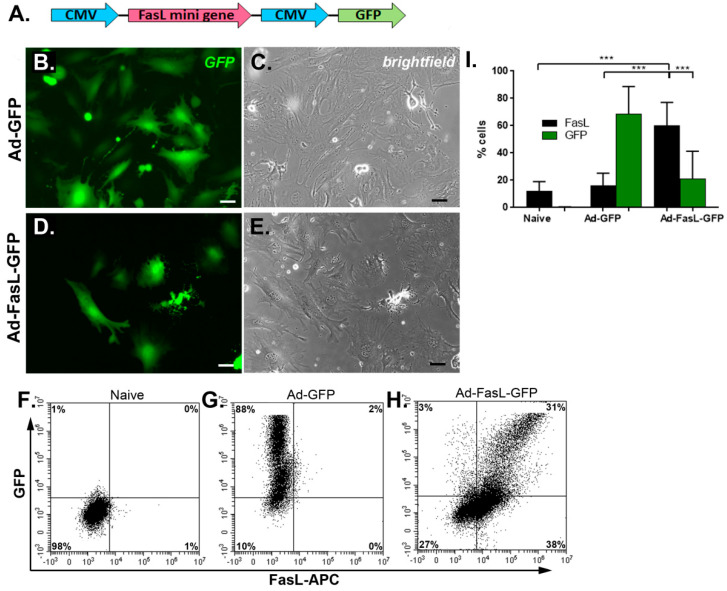
The transduction of MSC with Ad-FasL-GFP adenovirus-resulted in the overexpression of FasL. (**A**) A scheme depicting the vector used for the expression of the FasL protein coded by FasL truncated gene under the CMV promotor. Representative fluorescence (right) and brightfield (left) images of MSC transduced with an empty vector (Ad-GFP) (**B**,**C**) and FasL (Ad-FasL-GFP) (**D**,**E**) 48 h after transduction. Scale bar is 100 µm. Flow cytometry dot plots of naïve (**F**), Ad-GFP- (**G**) and Ad-FasL-GFP-transduced MSC (**H**). (**I**) Graph depicting the percentage of cells that express FasL in naïve, Ad-GFP- and Ad-FasL-GFP-transduced MSC 48 h after transduction. Data are means ± SD. *** *p* < 0.0005 by one-way ANOVA.

For the expression of FasL, a truncated sequence of the gene was inserted in the adenoviral expression vector under control of the cytomegalovirus (CMV) promoter alongside green fluorescent protein (GFP) as a reporter protein (Figure 1A,B). The FasL gene was modified to reduce its size by exclusion of the second intron from the murine FasL gene, as recently described [29]. The optimization steps showed the effective infection of MSC at 150 transduction units (TU)/cell, which were used for both the control (Ad-GFP) and the FasL gene-carrying adenovirus (Ad-FasL-GFP) (Figure 1). 

The MSC grown from the bone marrow of nonobese diabetic (NOD) mice displayed a slightly different morphology with a flatter and widespread structure than the regular fibroblast-like, spindle-shaped appearance of MSC derived from wild-type mice, as previously described [4,30]. The transduction with 150 TU/cell Ad-GFP and Ad-FasL-GFP did not alter the cell morphology and induced a strong cytosolic expression of the reported GFP protein in 68% ± 20% of cells transduced with Ad-GFP (Figure 1B,G,I and Supplementary Materials Figure S3B) and 21% ± 20% of cells transduced with Ad-FasL-GFP. The expression of FasL protein evaluated 48 h after transduction with an MLF4 antibody was detected in a small fraction (~2%) of naïve MSC and was present in 60% ± 17% of cells transduced with Ad-FasL-GFP (Figure 1H,I and Figure S3C). Notably, in MSC expressing FasL protein, less than half of the cells co-expressed the GFP reporter molecule (Figure 1H,I), suggesting a possible partial silencing of the second CMV promoter, as previously reported [29]

### 2.2. FasL Overexpression in MSC Does Not Impact Significantly Their Viability, Phenotype, Proliferation, Differentiation and Immunomodulatory Potential

**Figure 2:**
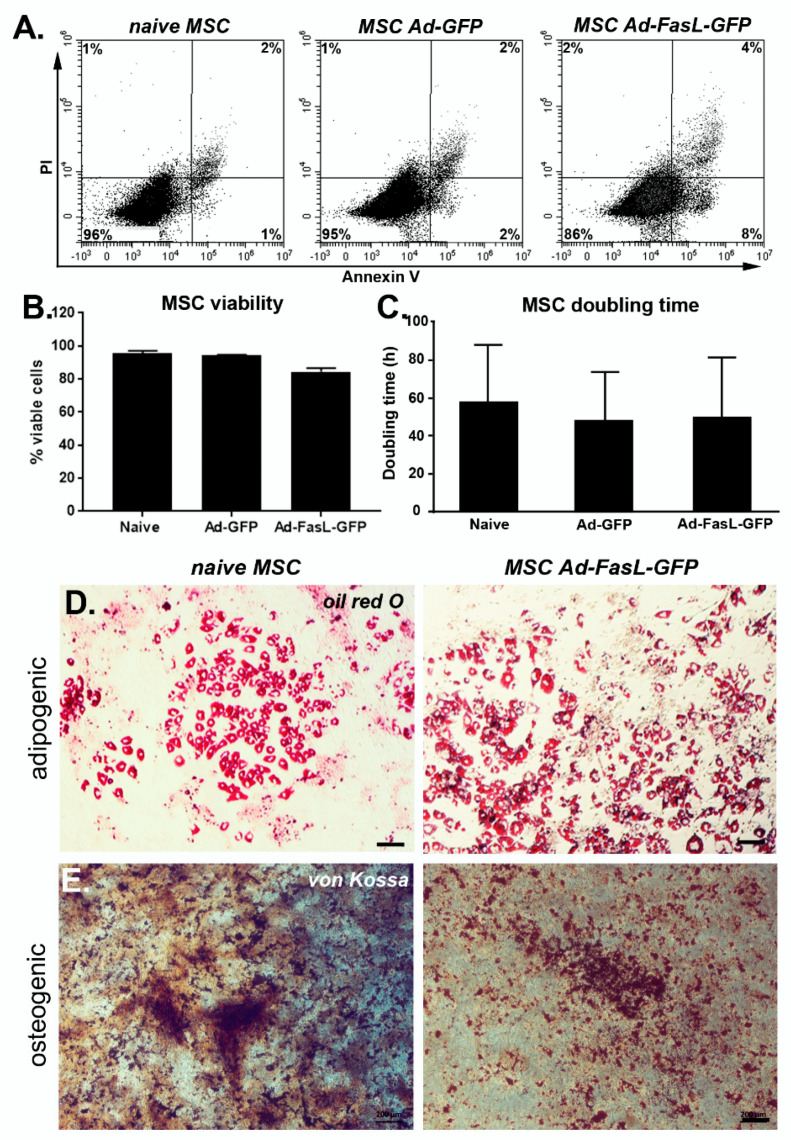

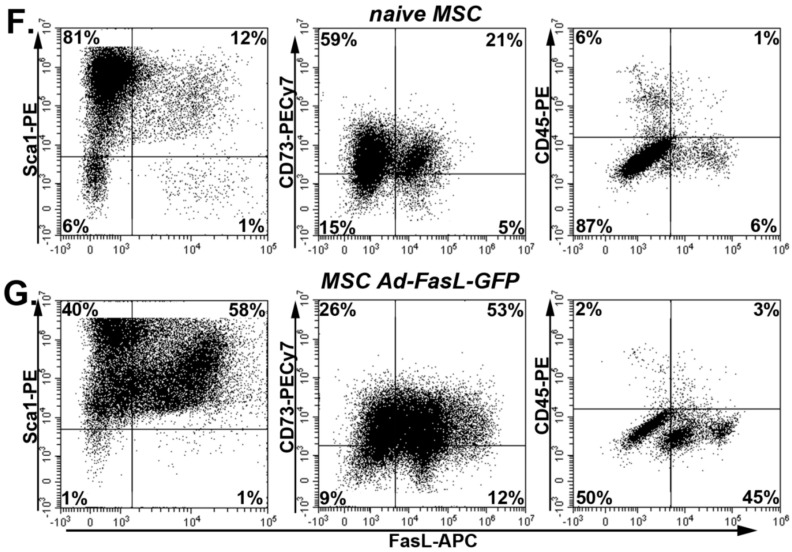
Transduction of MSC with FasL did not significantly affect the MSC patterns of behavior. (**A**) Representative flow cytometry dot plots of naïve, Ad-GFP and Ad-FasL-GFP-transduced MSC. Assessment of viability using Annexin V and propidium iodide (PI). (**B**) Summary of MSC viability at 24 h post-transduction. (**C**) Doubling time of naïve and transduced MSC from cultures at passages 5–8. Data are means ± SD. Representative images of naïve (left panels) and Ad-FasL-GFP-transduced MSC (right panels) that were incubated for 2 weeks in adipogenic induction medium and stained with oil red O (**D**) or in osteogenic differentiation medium and labeled with von Kossa staining (**E**), respectively. Scale bar is 100 µm. Expression of Sca1, CD73 and CD45 (y-axis) and FasL (x-axis) in naïve MSC (**F**) and MSC transduced with Ad-FasL-GFP (**G**).

To determine the impact of adenoviral infection, we assessed several specific characteristics of MSC isolated from the bone marrow of NOD mice. First, we evaluated the effect of MSC transduction with FasL on the cell viability. We found that the majority of the naïve bone marrow-derived MSC expressed low levels of Fas (Figure S1A); however, they are resistant to apoptotic signaling, as determined by exposure to toxic levels of FasL oligomers in culture (Figure S1C,D). While exposure to low concentrations of SuperFasL (5 ng/mL) had no influence on the cell viability, high doses of SuperFasL (25 ng/mL) induced apoptosis in ~10% ± 5% of the cells, as determined by Annexin V and propidium iodide (PI) incorporation (Figure S1C,D). MSC transduced to express the FasL protein displayed a slight decrease of ~16% ± 2% in viability, whereas MSC transduced with the control Ad-GPF virus sustained >95% viability (Figure 2A,B).

Second, the quantification of the doubling time of MSC in culture showed a negligible effect of either GFP or FasL expression, which was around 50 h (Figure 2C). Therefore, the transduction of MSC and overexpression of GFP and FasL did not induce significant apoptosis or attenuated the cell cycle rates. Third, MSC transduced to express FasL retained the potential to differentiate and adopt adipogenic and osteogenic phenotypes similar to naïve MSC (Figure 2D,E). Fourth, the presence of specific markers was monitored by flow cytometry, showing a prevalent expression of Sca1 (96% ± 4%), CD44 (~97%), CD105 (~92%) and CD73 (78% ± 3%) and the absence of c-kit (~0.6%) and CD45 (4% ± 4%) in naïve MSC grown in culture for 5–9 passages (Figure S2A–F and Figure 2F). Following the transduction with Ad-FasL-GFP, the levels of expression were 98% for Sca-1 and 79% for CD73, respectively, and was ~5% for CD45 expression (Figure 2G). Thus, the cell transduction and expression of GFP and FasL did not significantly change the MSC phenotype.

**Figure 3:**
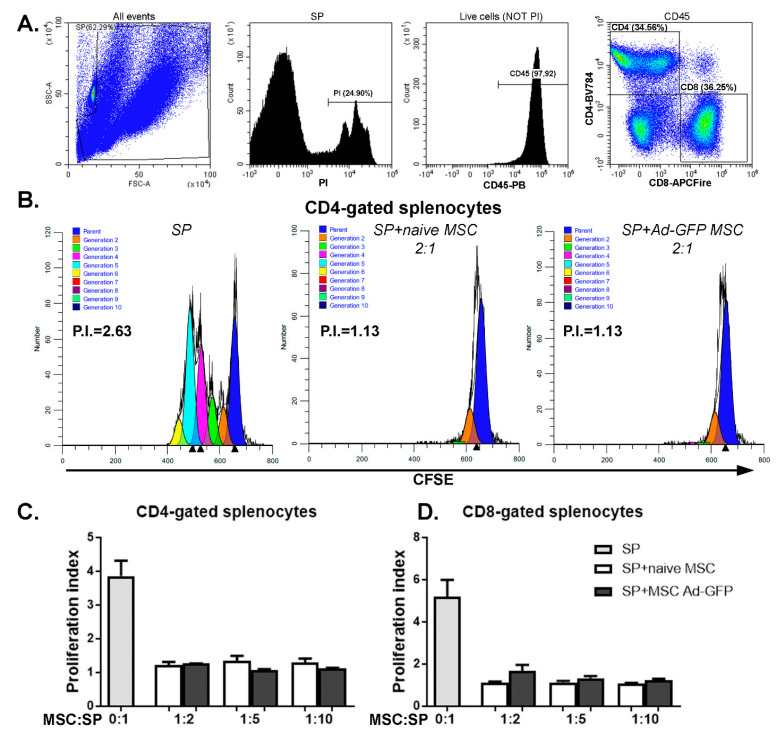
MSC transduced with Ad-GFP displayed immunomodulatory properties. (**A**) Gating strategy for assessing the proliferation of CFSE)-stained viable CD45CD4 and CD45CD8-activated splenocytes (SP). Proliferating CFSE-labeled cells were stained with PI and anti-CD45, -CD4 and CD8 antibodies. Splenocytes were gated out from the mix of cells and CD3/CD28 T cells activating beads to eliminate the beads from the analysis. Next, within the viable CD45-positive cell (negative for PI) population, CD4 and CD8-positive cells were determined. These populations were analyzed based on CFSE staining using ModFit software. (**B**) Representative ModFit proliferation plots of CD4-gated splenocytes that were cultured alone (SP), with naïve MSC (SP + naïve MSC) and with Ad-GFP-transduced MSC (SP + MSC Ad-GFP) in a ratio of 1:2 MSC to splenocytes. Graphs depicting the proliferation index of CD4- (**C**) and CD8-gated splenocytes (**D**) grown in the absence or presence of naïve MSC or MSC transduced with Ad-GFP for 72 h while stimulated with CD3/CD28 T cell activation beads.

Fifth, one of the particular properties of MSC is immunomodulation, often assessed by the proliferation rates of immune cells in mixed lymphocyte reactions (MLR). Effector MSC were co-incubated with CD3/CD28-stimulated splenocyte responders. The proliferation of gated CD8 T cells within the splenocyte preparation as assessed with a dilutional dye increased from 1.16 ± 0.09 to 5.93 ± 2.23 upon CD3/CD28 stimulation (Figure S2G,K). The co-incubation with naïve MSC inhibited T cell proliferation, starting at an effector-to-responder ratio of 1:20 (*p* < 0.005, 2.72 ± 1.08), and the inhibition increased at higher ratios (Figure S2G,K). Comparable results were obtained for CD4 T cells (Figure S2J), though the inhibition of proliferation was generally less efficient. A coculture of splenocytes with Ad-GFP-transduced MSC resulted in a similar suppression of both CD4 and CD8 T cells for all the ratios considered (Figure 3).

Altogether, these data indicate that MSC transduction to express either GFP or FasL did not significantly affect the viability, phenotype, cycling rates, differentiation potential or the suppression capacity of these cells.

### 2.3. MSC Overexpressing FasL Induce Apoptosis in Sensitive Cells

**Figure 4:**
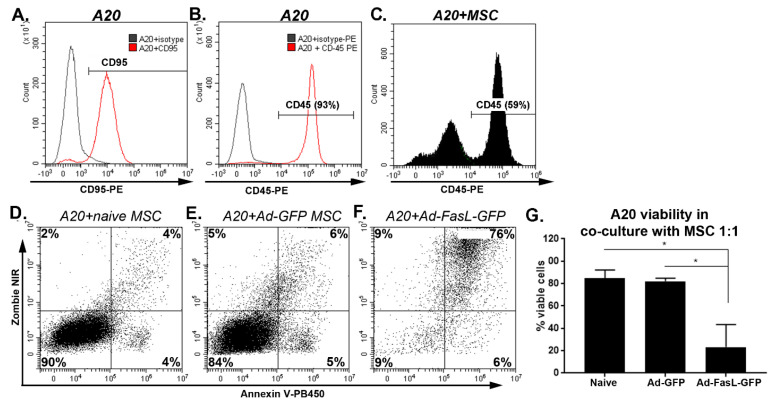
MSC overexpressing FasL induced a robust apoptosis of A20 cells. Expression of CD95 (**A**) and CD45 (**B**) in A20 cells by flow cytometry. (**C**) Gating on the CD45+ A20 cells in cocultures with MSC. (**D**) Apoptosis and death of gated A20 cells, as determined from Zombie NIR and Annexin V uptake after 24 h of coculture with naïve (**D**) and Ad-GFP- (**E**) and Ad-FasL-GFP-transduced MSC (**F**) at an A20:MSC ratio of 1:1. (**G**) Summary of A20 cell viability after a coculture with naïve and transduced MSC. Data are means ± SD (*n* = 2). * *p* < 0.05 by one-way ANOVA.

The overexpression of the FasL protein in MSC aims to endow these cells with killing activity. This property was evaluated in cocultures using, as targets, apoptosis-sensitive murine A20 leukemia/lymphoma cells, which express high levels of the Fas receptor (Figure 4A). The A20 cells, identified by CD45 expression (Figure 4B,C), were cocultured with naïve MSC and with MSC transduced with Ad-GFP and Ad-FasL-GFP, respectively (Figure 4C–F); 24 h later, the viability of CD45-gated A20 cells was maintained at 85% ± 7% and 82% ± 3%, respectively (Figure 4C–E,G). In marked contrast, the transduction of MSC to express FasL (with an average yield of 60% FasL expression, Figure 1) reduced the viability to 23% ± 20% of the target A20 cells, with >75% co-staining for both Annexin V as an apoptotic marker and for Zombie NIR as a cell death reporter. These data demonstrate that the FasL overexpressed by MSC was fully functional and conferred killer properties to MSC (Figure 4F,G).

**Figure 5:**
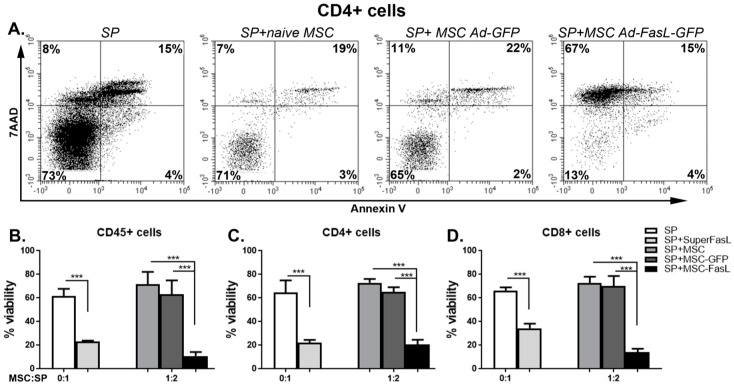
MSC overexpressing FasL induced the death of CD4 and CD8 T cells. (**A**) Freshly harvested splenocytes were activated with CD3/CD28 beads and cultured with or without adenovirus-infected MSC. After 72 h, the viability of different populations of splenocytes (CD45, CD4 and CD8) was determined. Representative dot plots of CD4-positive-activated splenocytes were cultured for 72 h in the absence (SP) or in the presence of naïve MSC (SP + naïve MSC), MSC transduced with Ad-GFP (SP + MSC Ad-GFP) and MSC transduced with Ad-FasL-GFP (SP + MSC Ad-FasL-GFP) at the ratio of 1:2 MSC to splenocytes and stained with 7-Aminoactinomycin D (7AAD) and Annexin V. Graphical representation of the data that shows the percentage of viable cells within the CD45 (**B**), CD45CD4 (**C**) and CD45CD8 (**D**)-positive cells, respectively. Data are means ± SD (*n* ≥ 3), *** *p* < 0.0005 by one-way ANOVA.

The efficacy of killer MSC overexpressing FasL in therapeutically relevant settings was tested by a coincubation for 72 h with CD3/CD28-stimulated splenocytes isolated from NOD mice (Figure S3). T cells identified by CD4+ and CD8+ expression in the whole splenocyte population showed remarkable and significant changes in cell death, from 27% ± 8% and 27% ± 12% death, respectively, following the coculture with naïve MSC to 79% ± 10% and 86% ± 7% death, respectively, following the incubation with MSC expressing FasL (Figure 5). These results were further substantiated by our findings regarding the viability of naïve and Ad-GFP-transduced MSC after the coculture with A20 cells and with CD3/CD28-activated splenocytes, respectively. The fluorescent images of the MSC in Figure S3, as well as the dot plots in Figure S5, revealed a similar viability of both naïve and Ad-GFP-transduced MSC, meaning that neither the A20 cells nor activated splenocytes were cytotoxic for the MSC.

## 3. Discussion

Mesenchymal stromal cells are endowed with great therapeutic potential, particularly in the inhibition of aberrant immune reactions. A potential use as immune suppressors raises the question of the possible elimination of the pathogenic cells. Therefore, we explored a strategy to enhance the capacity of MSC to kill activated immune cells by the overexpression of a death ligand. MSC were transduced with an adenovirus serotype 5 vector that is effective in gene delivery to slow cycling cells. In addition, the adenoviral DNA resides as episomes in the nucleus, and expression of the transgene is limited in time (for approximately three weeks), resulting in transient expression that is sensitive to cell-silencing mechanisms [25]. We found that Ad-FasL-GFP-transduced MSC overexpress FasL at least 10 days after transduction in vitro, which is important for the therapeutical perspective (Dumitrescu M., Institute of Cellular Biology and Pathology “Nicolae Simionescu”, Bucharest, Romania; unpublished work, 2020)).

The transduction of MSC to express GFP and FasL had an insignificant impact on the cell morphology, viability, proliferation and multilineage differentiation potential. Although MSC from NOD mice present a flatter morphology than the fibroblast-like appearance specific for MSC from wild-type mice, the transduction with Ad-FasL-GFP did not induce significant changes. Likewise, adenoviral transduction had no effect on the expression of characteristic phenotypic markers such as Sca-1 and CD73 and the absent expression of CD45. We chose these markers as they are strongly expressed in the MSC derived from various mouse strains [16,18]. These markers were also used to select MSC populations from the cocultures with A20 cells and splenocytes. The overexpression of either GFP and/or FasL had no significant impact on the MSC viability and doubling time in the culture, and the differentiation capacity to adopt adipogenic and osteogenic differentiation characteristics was not significantly modified. A recent study that focused on the effects of adenoviral transduction on the differentiation potential of human MSC showed that cells do not suffer major modifications in their behaviors unless the viral titers were very high. In the latter case, their adipogenic differentiation was impaired. This was also associated with lower cell proliferation rates [31]. These differences may rely on species specificity but, also, on the amount of virus used, as it is hard to estimate how the multiplicity of infection was calculated.

The NOD-derived MSC showed immunomodulatory properties revealed by a significant decrease of the proliferation index of both CD4 and CD8 T cells starting at a ratio of 1:20 MSC to splenocytes. Moreover, the transduction of MSC with Ad-GFP did show similar suppression properties as the naïve MSC on both CD4 and CD8 T cells at any of the ratios of MSC:splenocytes that we assessed.

FasL is a common executioner of apoptosis in the immune system. FasL could induce the death of activated B and T lymphocytes only in a membrane-bound form when its interaction with its cognate receptor, Fas, initiates the intracellular signaling cascade of apoptosis. The functionality of the transduced MSC to overexpress FasL was verified by the induction of Fas/FasL-induced apoptosis upon co-incubation with A20 cells, a mouse B lymphoma cell line that expresses Fas. Additionally, we assessed the induction of apoptosis of A20 cells after exposure to MSC transduced with either the adenovirus bearing only the reporter gene, the GFP, and the one that additionally carries the FasL gene. Our results showed that the apoptosis of A20 co-incubated with MSC transduced with Ad-FasL-GFP was significantly higher than in the case of A20 co-incubated with naïve and Ad-GFP-transduced MSC.

Yu and collaborators showed that increasing the FasL expression by knocking down microRNA Let-7a in bone marrow-derived MSC could induce CD3 T-cell apoptosis, both in vitro and in vivo, these cells proving more potent in the treatment of graft versus host disease and experimental colitis in murine models [32]. To test these novel “killer” MSC in a more therapeutically relevant setting, we evaluated their immunosuppressive properties by the capacity of inducing cell death to splenocytes isolated from NOD mice. These mice developed autoimmune diabetes spontaneously, and their immune cells represented a good and relevant therapeutic target. The CD3/CD28 activation of splenocytes induces the expression of Fas on their surface (Figure S4A–C), thus making them more prone to FasL-induced cell death. The exposure of activated splenocytes to SuperFasL resulted in substantially increased staining for Annexin V and PI than in the controls, suggesting that these cells are sensitive to FasL (Figure S4D,E). We found that MSC overexpressing FasL induced significant cell death of the majority of CD45-, CD4- and CD8-activated splenocytes upon coculture. Thus, the apoptosis induced by FasL signaling that corresponds to Ad-FasL-GFP-transduced MSC is superior to the immunomodulatory effects of naïve and Ad-GFP-transduced MSC. The immunosuppression mechanism of these novel “killer” MSC is enhanced, as the effect is more potent and more visible because it occurs faster than the proliferation of T cells, these being killed before attempting to proliferate.

Taken together, our data demonstrate that the NOD-sourced MSC that are modified via the adenoviral transfer of FasL are potent “killer” MSC exhibiting higher immunosuppressive potential without the loss of their main defining features. These results suggest that the FasL-overexpressing MSC could be a novel therapeutic tool to be employed in the fight with autoimmune disease like, for example, type 1 diabetes. Future research focused on identifying the optimal site of delivery of “killer” MSC, as well as the frequency of treatment administration, will define the outcome of the treatment.

## 4. Materials and Methods

### 4.1. Mice

The NOD/ShiLtJ (NOD) mice were purchased from The Jackson Laboratory and grown in our animal facility under specific pathogen-free conditions. All animals had access to food and water ad libitum. Blood glucose of NOD females was monitored in tail blood samples at weekly intervals using a glucometer (Roche Diagnostics, Florence, SC, USA) starting at 10 weeks of age. Diabetes was defined as two consecutive blood glucose measurements above 200 mg/dL. All the procedures were conducted in accordance with the European Guidelines for Animal Welfare and were approved by the Institutional Ethical Committee of the Institute of Cellular Biology and Pathology “Nicolae Simionescu”, from Bucharest, Romania and by the National Sanitary Veterinary and Food Safety Authority (authorization no. 296/23.08.2016).

### 4.2. MSC Isolation and Culture

MSC were isolated from bone marrow extracted from femurs and tibias of 8-week-old NOD male mice. After being passed through a 40-µm strainer, the cell aspirate was pelleted and resuspended in MSC media (DMEM low-glucose (LG) 1-g/L glucose (ThermoFisher, Waltham, MA, USA), 10% fetal bovine serum (FBS) suitable for MSC culture (MSC-FBS, PAN-Biotech, Wimborne, UK) and 1% penicillin/streptomycin/amphotericin (PSA, Sigma, St. Louis, MO, USA) and cultured starting with a 2 × 10^6^ cells/cm^2^ cell density. Cells were left to adhere for 24 h; then, media was replaced every 2 to 3 days. The adherent cells were left to reach confluency and then subjected to several passages, each time at a 1:4 split. Cells were grown in a 37 °C, 5% CO_2_ humidified incubator. All the MSC in this study were used between passage 5 and 9.

### 4.3. Viral Transduction of MSC

Transduction of MSC was optimized for a smaller amount of virus to infect a larger number of cells. To achieve that, MSC were detached by 0.125% trypsin-Ethylenediaminetetraacetic acid (EDTA) (Sigma, St. Louis, MO, USA), washed and resuspended in DMEM 1-g/L glucose without sodium bicarbonate (Sigma, St. Louis, MO, USA) supplemented with 10% MSC-FBS at a density not larger than 2.5 × 10^6^ cells/mL. Maximum 200-µL cell suspension (5 × 10^5^ cells) was added in a 1.5-mL low-retention Eppendorf tube, followed by the addition of the corresponding amount of virus. The tubes were then stirred at 1000 rpm for 45 min at 37 °C on a thermomixer, without allowing the cells to sediment. Cells were seeded at corresponding densities, depending on the following experiments.

### 4.4. Osteogenic and Adipogenic Differentiation of MSC

To determine the multipotency of MSC overexpressing FasL, cells transduced as described above and were seeded at a 5000 cell/cm^2^ in 24-well plates and left to adhere overnight. Twenty-four hours after transduction, media was changed with adipogenic and osteogenic induction media, respectively, as well as normal MSC media. Media was changed every 2 to 3 days. The adipogenic induction media contained DMEM 1-g/L glucose supplemented with 10% MSC-FBS, 10^−6^-M dexamethasone, 100-mM indomethacin and 1% insulin-transferrin-selenium (ITS-G, ThermoFisher, Waltham, MA, USA), and the osteogenic induction media was based on DMEM 1-g/L glucose supplemented with 10% MSC-FBS, 10^−7^-M dexamethasone, 10-mM β-glycerophosphate and 0.3-mM ascorbic acid. After 2 weeks, cells were washed with phosphate buffer saline (PBS), fixed with 4% paraformaldehyde and stained with Oil Red O to visualize the lipid droplets accumulated in the differentiated adipocytes or with von Kossa stain represented by incubation in 5% AgNO_3_, followed by a short 2-min rinse with 5% sodium thiosulphate to visualize the Ca^2+^ crystals. All the images were taken using an Olympus CKX41 microscope with an Olympus XC30 camera and minimally processed using Adobe Photoshop software.

### 4.5. Flow Cytometry Analysis

For flow cytometric analysis, MSC were stained with the following antibodies: anti-mouse Sca1_PE_, anti-mouse CD73_PE/Cy7_, anti-mouse CD117_APC_ (c-kit), anti-mouse CD45_PE_, anti-mouse CD44_PE_, anti-mouse CD95_PE_ (Biolegend, San Diego, CA, USA), goat anti-mouse CD105 and the secondary rabbit anti-goat_FITC_ (Invitrogen, Carlsbad, CA, USA). The antibody mix that was used to label the splenocytes contained anti-mouse CD45_PE_, anti-mouse CD4_BV784_ and anti-mouse CD8a_APCfire_ (Biolegend, San Diego, CA, USA). FasL was detected using anti-FasL_AF647_ antibody (MLF4 clone, Bio-Rad, CA, USA). For all the antibodies, we used the corresponding isotypes from the same sources. Samples were analyzed using a Cytoflex flow cytometer (Beckman Coulter, Indianapolis, IN, USA) and CytExpert software. Determination of the proliferation index of the splenocytes was done based on the carboxyfluorescein succinimidyl ester (CFSE) readings that were processed using ModFit LT^TM^ software (Verity Software House, Topsham, ME, USA). For the CFSE analysis, the unstimulated splenocytes were used as the parent (basal fluorescence).

### 4.6. Immunosuppression Assay

Detection of FasL-induced apoptosis and cell death was done upon coculturing MSC with A20 and with freshly isolated splenocytes. The two methods will be detailed below.

#### 4.6.1. Functional Characterization of MSC Overexpressing FasL Using A20 Cells

MSC were transduced as described above and left to adhere in MSC media. Twenty-four hours later, media was removed, and A20 cells were added in a ratio of 1:1 and incubated for another 24 h. After this time, all the cells were collected; stained with anti-CD45 antibody, Zombie NIR and Annexin V_PB450_ (Biolegend, San Diego, CA, USA) and analyzed by flow cytometry. We gated cells on the CD45-positive population and evaluated the percent of viable cells by excluding the cells stained with Zombie NIR (dead cells) and Annexin V (apoptotic).

#### 4.6.2. Death and Suppression Assay for MSC Overexpressing FasL Using Whole Splenocytes Cells Obtained from NOD Mice

NOD female mice, characterized by two different glycemia readings above 200 mg/dL within a week, were the source of the splenocytes. Freshly harvested cells were resuspended in RPMI medium supplemented with 10% FBS, 1% PSA and 50-µM β-mercaptoethanol (MLR medium) in the presence or absence of CD3/CD28 stimulation beads (in a ratio of 1:1 beads-to-splenocytes) (Dynabeads mouse T-Activator CD3/CD28, Gibco). Splenocytes both unstimulated and stimulated were stained with 2.5-μM CFSE (a dilutional dye) and were cultured alone or in the presence of naïve, Ad-GFP- and Ad-FasL-GFP-transduced MSC (as above) in a ratio of 1:2 MSC-to-splenocytes for 72h in the MLR medium. At the end of the coincubation, splenocytes were carefully detached from the MSC by gentle rinsing using a 0.05-mM EDTA in PBS solution stained with anti-CD45, -CD4 and -CD8 antibodies; PI/ 7-Amino Actinomycin D (7AAD) and Annexin V_APC_ and analyzed by flow cytometry. SUPERFASLIGAND^®^ (SuperFasL) protein (Enzo Life Sciences, Farmingdale, NY, USA) was used as a control for FasL-induced apoptosis.

### 4.7. Determination of Doubling Time

The impact of GFP or FasL expression on MSC proliferation was assessed by using an xCelligence Real-Time Cell Analysis (RTCA) DP instrument (Roche, Basel, Switzerland). Twenty-four hours after transduction with Ad-GFP and Ad-FasL-GFP, naïve or transduced MSC were plated in a microplate and monitored for 72 h. Doubling time was determined using RTCA software.

### 4.8. Statistical Analysis

Analysis of the data was performed using the GraphPad Prism program (GraphPad Software, San Diego, CA, USA). Data are presented as ± SEM or SD and analyzed with the statistical tests indicated in the text and in the figure legends. Samples that had a *p*-value < 0.05 were considered statistically significant, and when *p* was not indicated, samples were not significantly different.

## Data Availability

Data are contained and available within this manuscript.

## Supplementary Materials

Supplementary Materials can be found at https://www.mdpi.com/1422-0067/22/1/348/s1.

## Abbreviations

FasL	First apoptosis signal ligand
BM-MSC	Bone marrow-derived mesenchymal stromal cells
GFP	Green fluorescent protein
PI	Propidium iodine
NOD mice	Nonobese diabetic mice
CFSECFSE	carbocarboxyfluorescein succinimidyl ester
