# Supplementary material for: Enhanced Suppression of Immune Cells In Vitro by MSC Overexpressing FasL
Source: Int J Mol Sci. 2020 Dec 31;22(1):348. doi: 10.3390/ijms22010348 (PMC7795906; doi:10.3390/ijms22010348)
Supplement: Supplementary file 1 [file ijms-22-00348-s001.pdf]

International Journal of *Molecular Sciences*

MDPI

Supplementary Table S1: Circulating Exosomal miRNAs as Biomarkers for the Diagnosis and Prognosis of Colorectal Cancer.

Katiusse Alves dos Santos, Isabelle Cristina Clemente dos Santos, Carollyne Santos Silva, Hériks Gomes Ribeiro, Igor de Farias Domingos and Vivian Nogueira Silbiger.

Supplementary Table S1. Main circulating exosomal miRNAs involved in CRC.

Authors	Casuistic	Upregulated	Downregulated
Karimi et al., 2019	25 patients with CRC and 13 healthy controls	miR-301a and miR-23a	
Liu et al., 2018	369 individuals	miR-27a and miR-130a	[1]miR-27a and miR-130a
Ogata-Kawata et al., 2014	88 CRC patients with primary tumors, 29 patients after primary tumor resection surgery and 11 healthy controls	let-7a, miR-1224-5p, miR-1229, miR-1246, miR-1268, miR-1290, miR-1308, miR-150, miR-181b, miR-181d, miR-1915, miR-21, miR-223, miR-23a, miR-483 -5p, and miR-638	[2]let-7a, miR-1224-5p, miR-1229, miR-1246, miR-150, miR-21, miR-223 and miR-23a
Zhu et al., 2017	196 patients with CRC and 138 healthy individuals	miR-19a-3p, miR-21-5p and miR -425-5p	
Ren et al., 2017	150 patients with CRC and 90 healthy controls	miR-196b-5p	
Cheng et al., 2019	53 CRC patients	miR-146a	
Wang et al., 2017	50 patients in stages I and II and 50 healthy individuals	miR-125a-3p and miR-320c	
Zhang et al., 2019	18 patients with CRC and 18 healthy controls	miR-17-5p, miR-181a-5p, miR-18a-5p and miR-18b-5p	

Min et al., 2019	114 patients (40 CRC, 22 adenoma and 52 controls)		miR-92b
Liu et al., 2018	10 CRC patients pre and postoperative	miR-486-5p	[3]miR-486-5p
Takano et al., 2017	240 patients with CRC	miR-203	
Yan et al., 2017	77 patients with CRC and 20 healthy controls	miR-486-5p and miR-3180-5p	miR-638, miR-5787, miR-8075, miR-6869-5p and miR-548c-5p
Yan et al., 2018	168 patients with CRC and 20 healthy controls	miR-6803-5p	
Yan et al., 2018	142 patients with CRC and 40 healthy controls		miR-6869-5p
Fu et al., 2018	18 patients with CRC, 11 patients with metastasis, and 10 normal controls	miR-17-5p and miR-92a-3p	
Peng; Gu; Yan., 2019	108 patients with CRC		miR-548c-5p
Tang et al., 2019	34 patients with metastatic CRC and 108 non-metastatic	miR-320d	
Yu et al., 2017	411 patients with CRC, 58 patients with adenoma, 175 healthy and 10 pre- and post-chemotherapy patients	[4]miR-217	miR-217
Tsukamoto et al., 2017	326 patients with CRC according to the TNM stage (51 stage I, 110 stage II, 98 stage III and 67 stage IV)	miR-21	

Shao et al., 2018	80 healthy controls, 30 patients with adenoma, 65 patients with CRC with liver metastasis and 60 patients with CRC without liver metastasis	miR-21	
Santasusagna et al., 2018	50 patients with CC stage I-III		miR-200c and miR-141
Matsumura et al., 2015	227 CRC patients and 28 healthy controls	miR-19a, miR-19b, miR-23a, miR-92a, miR-320a and miR-4437	
Liu et al., 2016	84 participants with stage II / III CC after tumor resection and before adjunctive therapy	miR-4732-5p	miR-4772-3p
Li et al., 2017	85 patients with CRC stage III		miR-96-5p and miR-149
Monzo et al., 2017	50 patients undergoing CC stage I to III surgery	let-7g, miR-15b, miR-155 and miR-328	
Bjørnetrø et al., 2019	24 patients with LARC	miR-30d-5p	miR-486-5p and miR-181a-5p
Jin et al., 2019	43 patients with CRC stages III and IV	miR-21-5p, miR-96-5p, miR-1246 and miR-1229-5p	
Yagi et al., 2019	3 patients with CRC and stable disease, 3 with progressive disease, 3 healthy volunteers and 55 patients with advanced/recurrent CRC	miR-125b	

CRC - colorectal cancer; [1]Unregulated after surgical resection; [2]Reduction of expression levels after surgical resection of primary tumor; [3]Unregulated after surgical resection; [4]Regulated upward after chemotherapy; CC - Colon cancer; LARC - Locally Advanced Rectal Cancer; TNM - Classification of Malignant Tumours.
